# *SPG7* mutations in amyotrophic lateral sclerosis: a genetic link to hereditary spastic paraplegia

**DOI:** 10.1007/s00415-020-09861-w

**Published:** 2020-05-23

**Authors:** Alma Osmanovic, Maylin Widjaja, Alisa Förster, Julia Weder, Mike P. Wattjes, Inken Lange, Anastasia Sarikidi, Bernd Auber, Peter Raab, Anne Christians, Matthias Preller, Susanne Petri, Ruthild G. Weber

**Affiliations:** 1 Department of Human Genetics, Hannover Medical School, Carl-Neuberg-Straße 1, 30625 Hannover, Germany; 2 Department of Neurology, Hannover Medical School, Hannover, Germany; 3 Institute for Biophysical Chemistry, Hannover Medical School, Hannover, Germany; 4 Department of Diagnostic and Interventional Neuroradiology, Hannover Medical School, Hannover, Germany

**Keywords:** Motor neuron disease, Amyotrophic lateral sclerosis, Hereditary spastic paraplegia, Whole-exome sequencing, SPG7

## Abstract

Amyotrophic lateral sclerosis (ALS) and hereditary spastic paraplegia (HSP) are motor neuron diseases sharing clinical, pathological, and genetic similarities. While biallelic *SPG7* mutations are known to cause recessively inherited HSP, heterozygous *SPG7* mutations have repeatedly been identified in HSP and recently also in ALS cases. However, the frequency and clinical impact of rare *SPG7* variants have not been studied in a larger ALS cohort. Here, whole-exome (WES) or targeted *SPG7* sequencing was done in a cohort of 214 European ALS patients. The consequences of a splice site variant were analyzed on the mRNA level. The resulting protein alterations were visualized in a crystal structure model. All patients were subjected to clinical, electrophysiological, and neuroradiological characterization. In 9 of 214 (4.2%) ALS cases, we identified five different rare heterozygous *SPG7* variants, all of which were previously reported in patients with HSP or ALS. All detected *SPG7* variants affect the AAA+ domain of the encoded mitochondrial metalloprotease paraplegin and impair its stability or function according to predictions from mRNA analysis or crystal structure modeling. ALS patients with *SPG7* mutations more frequently presented with cerebellar symptoms, flail arm or leg syndrome compared to those without *SPG7* mutations, and showed a partial clinical overlap with HSP. Brain MRI findings in *SPG7* mutation carriers included cerebellar atrophy and patterns suggestive of frontotemporal dementia. Collectively, our findings suggest that *SPG7* acts as a genetic risk factor for ALS. ALS patients carrying *SPG7* mutations present with distinct features overlapping with HSP, particularly regarding cerebellar findings.

**Electronic supplementary material:**

The online version of this article (10.1007/s00415-020-09861-w) contains supplementary material, which is available to authorized users.

## Introduction

Amyotrophic lateral sclerosis (ALS) and hereditary spastic paraplegia (HSP) belong to the etiologically heterogeneous group of motor neuron diseases (MNDs) characterized by progressive degeneration and functional decline of motor neurons [1]. In addition to signs of motor neuron involvement, both ALS and HSP patients may exhibit extra-motor symptoms. Up to 50% of ALS patients develop cognitive and behavioral impairment, which fulfill the criteria of frontotemporal dementia (FTD) in up to 15% of cases [2]. Extra-motor symptoms in so-called “pure” or “uncomplicated” HSP patients are urge incontinence and mild pallhypaesthesia. In “complicated” or “complex” HSP, patients may additionally develop cerebellar dysfunction (ataxia, nystagmus, tremor), peripheral neuropathy, cognitive impairment, extrapyramidal features, and ophthalmological abnormalities [3]. Despite remarkable progress in understanding the molecular mechanisms of both ALS and HSP, no effective treatment currently exists [1]. The discovery of an increasing number of causative gene mutations results in deeper insights into the etiology of both diseases and might enable identification of future therapeutic targets. Interestingly, mutations in several genes such as *VCP*, *SPG11*, and *KIF5A* were reported to cause ALS and HSP [4–6], highlighting shared genetic mechanisms of clinically distinct MNDs.

Here, whole-exome sequencing (WES) in a pilot cohort of 23 ALS patients to detect rare ALS-associated variants yielded recurrent mutations in an HSP-associated gene, *SPG7*. We then aimed at systematically studying the frequency, predicted consequences on the protein level, and clinical impact of *SPG7* mutations in a larger European ALS cohort.

## Patients and methods

### Subjects and their clinical evaluation

The study was approved by the Ethics Board of Hannover Medical School (ID # 6269). Written informed consent was obtained from all patients. The study cohort consisted of 214 European patients (126 males, 88 females) diagnosed with ALS [6 familial ALS (fALS), 208 sporadic ALS (sALS) cases] based and recruited at the ALS/MND Clinic of the Department of Neurology of Hannover Medical School, Germany. Patients were subdivided into one of eight clinical subtypes [7]: (1) upper motor neuron (UMN) dominant ALS: predominant UMN involvement and clinical/neurophysiological signs of lower motor neuron (LMN) affection during follow-up; (2) bulbar phenotype (B): bulbar onset and predominant but not exclusive bulbar affection throughout the disease course; (3) flail arm syndrome: progressive, predominantly proximal, symmetric weakness and wasting in the upper limbs with little or no functional impairment of the bulbar muscles or legs 12 months after symptom onset; (4) flail leg syndrome: isolated weakness and wasting in the lower limbs for 12–24 months after symptom onset; (5) respiratory phenotype: predominant respiratory impairment at onset and only mild spinal or bulbar signs for 12 months; (6) progressive muscular atrophy (PMA): progressive LMN involvement and no clinical UMN signs; (7) LMN dominant ALS: predominant LMN symptoms with UMN signs at some point in the disease; (8) classic (Charcot’s) ALS: LMN and UMN signs in more than two regions within a period of 12 months after symptom onset. Disease progression was evaluated using the revised version of the ALS functional rating score (ALSFRS-R). At first presentation, the progression rate was calculated using the following formula: 48 − total ALSFRS-R/symptom duration in months [8]. Neurocognitive or behavioral impairments were assessed using the Edinburgh Cognitive and Behavioural ALS Screen (ECAS) [9] or the frontal assessment battery (FAB) [10] in 32 patients, and based only on clinical impression in 182 patients. Clinical cerebellar dysfunction was diagnosed if symptoms of cerebellar ataxia including gait ataxia and intention tremor in extremities not affected by weakness or oculomotor disturbances (saccadic pursuit, nystagmus) were present.

### DNA, RNA, and protein analyses

Genomic DNA was extracted from whole blood using the QIAamp DNA Blood Kit (Qiagen, Hilden, Germany). WES was performed on DNA from 23 ALS patients using Agilent SureSelect Human All Exon v4 Target Enrichment System on an Illumina HiSeq 2000 by Oxford Gene Technology, Begbroke, UK, as described previously [11]. All samples were sequenced to a mean target coverage of > 50×. WES data were analyzed using our in-house workflow based on INGENUITY Variant Analysis (QIAGEN Bioinformatics, Redwood City, CA, USA). To screen all exons (1–17) and flanking intronic sequences of *SPG7* (NM_003119.2) for variants in 191 further ALS patients and to verify selected *SPG7* variants identified by WES, conventional chain termination protocols were used. Primers and amplification conditions were adapted from a previously published study [12]. Minor allele frequencies (MAF) of genetic variants in non-Finnish Europeans were extracted from the ExAC Browser Beta database (https://gnomad.broadinstitute.org/). Variant pathogenicity was predicted using SIFT according to Alamut Visual software, version 2.11 (Interactive Biosoftware, Rouen, France), PolyPhen-2 (http://genetics.bwh.harvard.edu/pph2/), and the American College of Medical Genetics (ACMG) criteria [13]. Potential effects on gene splicing were evaluated using Alamut Visual software. The Human Gene Mutation Database Professional 2018.1, the ALS Online Genetics Database ALSoD (https://alsod.ac.uk), and PubMed (https://pubmed.ncbi.nlm.nih.gov) were interrogated for previously reported *SPG7* variants.

Total RNA was extracted from whole blood of patient MD018 using the RNeasy Mini Kit (Qiagen), and cDNA was synthesized on total RNA using the SuperScript III First-Strand Synthesis Kit (Thermo Fisher Scientific, Waltham, MA, USA). Subsequently, cDNA was sequenced using conventional chain termination protocols.

Predicted amino acid alterations were introduced into the crystal structure of paraplegin (PDB: 2qz4) [14], and the mutated structures were energy minimized using Macromodel of the Schrödinger Suite and the OPLS3 force field [15, 16]. A structural model of the paraplegin hexamer was constructed by alignment on the hexameric FtsH complex structure (PDB: 2dhr).

### Neurophysiological studies and cranial magnetic resonance imaging (MRI)

All patients were examined by nerve conduction studies (NCS) and electromyography (EMG) to detect sensory/motor neuropathy and the extent of LMN affection. Multisequence brain MRI studies of seven *SPG7* mutation carriers done during diagnostic workup were reassessed by two neuroradiologists (MPW, PR) with regard to imaging findings associated with neurodegenerative diseases, such as corticospinal tract hyperintensities and global or regional cortical atrophies, particularly corpus callosum (CC) thinning and cerebellar atrophy [17–19]. The total midsagittal CC area was evaluated on T1- or T2-weighted sagittal MRI images, manually estimated using ROI analysis, and compared to anatomical references (normal range 580–1040 mm^2^) [20].

## Results

### Identification of rare heterozygous *SPG7* variants in 9 of 214 European ALS patients

**Fig. 1:**
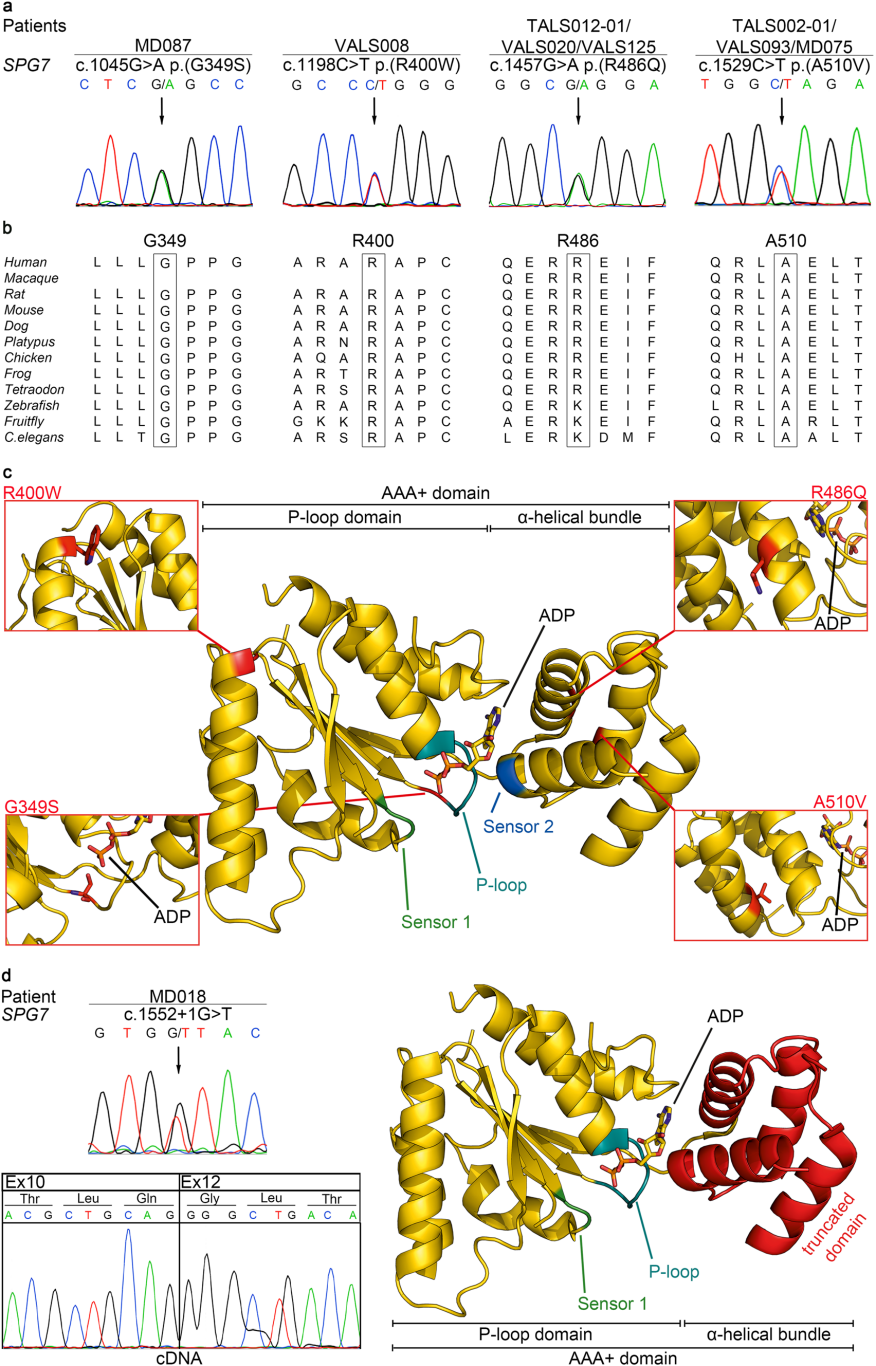
Five heterozygous *SPG7* mutations were detected in nine patients of a European ALS cohort. **a** Electropherograms demonstrating four rare heterozygous *SPG7* missense variants in DNA from whole blood of eight ALS patients (affected nucleotides are designated by an arrow). All variants were predicted to be deleterious by at least one of two prediction tools, i.e. SIFT according to Alamut Visual Version 2.11, Interactive Biosoftware, Rouen, France or PolyPhen-2. **b** Missense variants detected in *SPG7* affect amino acids highly or very highly conserved in paraplegin orthologs from different species (data taken from Alamut Visual Version 2.11). **c** Mapping of the amino acid residues affected by the identified *SPG7* missense variants on a structural model of the AAA+ domain of human paraplegin (gold) with its substrate adenosine diphosphate (ADP). Close-up views show the mutated residues as obtained after energy minimization using Macromodel of the Schrödinger Suite and the OPLS3 force field [15, 16]. Variant p.(G349S) is part of the P-loop and may affect nucleotide binding and hydrolysis. In contrast, p.(R486Q) and p.(A510V) may disturb the structure of the α-helical bundle and thereby oligomerization. p.(R400W) is located in the N-terminal P-loop domain and as part of the hexamer interface may interfere with formation of the supracomplex. Sensor 1 is shown in green, Sensor 2 in blue, and the P-loop (Walker A motif) in cyan. **d** The rare heterozygous *SPG7* splice site variant c.1552 + 1G > T detected in ALS patient MD018 on genomic DNA, its effect on cDNA, and its predicted consequence on the protein level. Given are electropherograms of targeted *SPG7* sequencing on genomic DNA and cDNA from whole blood of patient MD018. By cDNA sequencing, an aberrant *SPG7* transcript lacking exon 11 was identified. The predicted consequence of the aberrant transcript on the protein level was visualized in a crystal structure model of the functional AAA+ domain of paraplegin composed of the P-loop domain and the α-helical bundle. The skipping of exon 11 is predicted to result in a frameshift, leading to a premature stop codon. This would result in an altered α-helical bundle as indicated by the red color and a truncated peptidase domain

To detect rare ALS-associated variants, WES was performed on DNA from whole blood of 23 unrelated ALS cases, and variants with a MAF of < 1% in genes associated with ALS according to ALSoD (*n* = 126) that were classified as pathogenic or likely pathogenic using ACMG guidelines were retrieved from the datasets. This filtering strategy identified three variants, each of which was carried by one patient, in two genes, *SPG7* and *LIPC,* whereby *SPG7* was recurrently affected (Supplementary Table 1). Both rare *SPG7* variants, the missense c.1529C > T p.(A510V) variant in patient TALS002-01 and the splice site c.1552 + 1G > T variant in patient MD018, were heterozygous as confirmed by Sanger sequencing (Fig. 1a, d), and had previously been described in ALS or HSP patients (Table 1). To test the consequence of the splice site variant c.1552 + 1G > T, we amplified a part of *SPG7* comprising exons 10–12 on cDNA from whole blood of patient MD018. By cDNA sequencing, an aberrant *SPG7* transcript lacking exon 11 was identified (Fig. 1d). The altered transcript encoded by the c.1552 + 1G > T variant was predicted to result in an aberrant protein after amino acid 483 and a premature truncation after amino acid 556.

**Table 1:** Rare heterozygous non-silent variants in the *SPG7* gene predicted to be deleterious identified in 214 European ALS patients

Sample	Chromosomal position GRCh37/hg19	Exon	Nucleotide change	Amino acid change	dbSNP	MAF	Prediction tools		ACMG guidelines	Predicted consequence on protein level	Previously described in
							SIFT	PolyPhen-2			
MD087	16:89598369	8	c.1045G > A	p.(G349S)	rs141659620	0.001437	Deleterious	Probably damaging	Pathogenic	Effect on P-loop (Walker A motif), hence on nucleotide binding and potentially on hydrolysis (coupling of the hydrolysis state)	ALS [26], CA [37], HSP [28], UMN [24]
VALS008	16:89598918	9	c.1198C > T	p.(R400W)	rs748024868	–	Deleterious	Probably damaging	Likely pathogenic	Destabilization of N-terminal domain and supracomplex formation (mutation is part of the hexamer interface)	HSP [12]
TALS012-01 VALS020 VALS125	16:89613073	11	c.1457G > A	p.(R486Q)	rs111475461	0.007232	Deleterious	Benign	Uncertain significance	Similar effect as p.(A510V) possible. Destabilization of α-helical bundle and sensor 2	HSP [19]
TALS002-01 VALS093 MD075	16:89613145	11	c.1529C > T	p.(A510V)	rs61755320	0.004004	Deleterious	Probably damaging	Pathogenic	May sterically disturb interactions between helices 5 and 6, and destabilize the α-helical bundle, hence have an effect on opening/closing and complex formation, may also affect sensor 2 (aa518-520, GAP motif) that couples hydrolysis state to oligomerization state	ALS [27], CA [37], HSP [12, 19, 28], UMN [24]
MD018	16:89613169	11^a^	c.1552 + 1G > T	–	rs141644720	0.00006002	–	–	Pathogenic	Altered α-helical bundle (aa483-556[stop]), hence effect on opening/closing and supracomplex formation, peptidase domain is missing	HSP [18]

Reference sequence used: NM_003119.2. Only variants in the coding region of *SPG7* and canonical ± 1 or 2 splice site variants are given

All variants are located in the AAA+ domain (amino acid residues 305–565) [14] of paraplegin

*aa* amino acids, *ACMG* *guidelines* interpretation of sequence variants according to the American College of Medical Genetics and Genomics guidelines [13], *ALS* amyotrophic lateral sclerosis, *CA* cerebellar ataxia, *HSP* hereditary spastic paraplegia, *MAF* minor allele frequency in European (non-Finnish) population according to Exome Aggregation Consortium Browser (Beta), *SIFT* according to Alamut Visual version 2.11, *UMN* upper motor neuron syndrome

^a^Intron 11

To study the *SPG7* variant frequency in a larger ALS cohort, we performed mutational analysis of all 17 *SPG7* exons and adjacent splice site regions (± 20 base pairs) on whole blood DNA of 191 further ALS patients. We detected seven patients carrying four different rare heterozygous missense variants in *SPG7* predicted to be deleterious according to SIFT or PolyPhen-2 and affecting highly or very highly conserved amino acid residues (Fig. 1a, b and Table 1). All identified *SPG7* nucleotide changes were summarized in Supplementary Table 2.

Taken together, in 9 of 214 (4.2%) ALS patients we identified five different rare *SPG7* variants predicted to be deleterious, four missense and one essential splice site variant, all of which were heterozygous (Fig. 1a, d). None of the *SPG7* variant carriers harbored additional non-synonymous *SPG7* variants, excluding compound heterozygosity for pathogenic *SPG7* variants. All identified *SPG7* variants were previously described in HSP patients, and two of the missense variants were reported in ALS cases. Two *SPG7* variants were inherited from mothers (aged 75 and 76 years) not affected by ALS to date suggesting reduced penetrance.

### Crystal structure modeling of detected rare *SPG7* variants

The *SPG7* gene encodes paraplegin, a mitochondrial metalloprotease containing an AAA+ (ATPases associated with diverse cellular activities) domain that couples ATP hydrolysis to protein remodeling flanked by an FtsH-extracellular and a peptidase domain, which is thought to form a hexameric complex. All five different rare *SPG7* variants identified here in ALS patients clustered in exons 7–13 that code for the paraplegin AAA+ domain, i.e. amino acid residues 305–565 (Fig. 1c, d) [14]. Variant p.(G349S) is located in the N-terminal P-loop of the AAA+ domain. Glycine at position 349 is part of the Walker A motif with its consensus sequence GxxGxGK(T/S), involved in the nucleotide binding process and ATP hydrolysis. Variant p.(R400W) is part of the α3-helix in the P-loop domain. The exchange of the positively charged arginine by the bulky hydrophobic tryptophan might destabilize the N-terminal domain and the adjacent five-stranded β-sheet. As this variant maps to the interface between the AAA+ monomers (see Supplementary Fig. 1) and changes the surface properties, it may also have a destabilizing effect on the supracomplex formation. Variants p.(R486Q) and p.(A510V) map to the α-helical bundle domain within a radius of 3 Å. The slightly more bulky side chain of p.(A510V) might interfere with the interactions that stabilize α-helices 5 and 6, disturbing the opening and closing of the α-helical bundle domain, which is thought to be important for oligomerization to the supracomplex. Additionally, p.(A510V) could affect the nearby Sensor 2 motif that is assumed to play a role in coupling the hydrolysis state to the oligomerization state [14]. The close vicinity of p.(R486Q) and p.(A510V) indicates a similar effect of both variants on the stability and function of the α-helical bundle domain, Sensor 2, and the supracomplex formation. The splice site variant c.1552 + 1G > T leads to a transcript lacking exon 11 predicted to cause a frameshift and a premature stop codon [18] that would alter the entire α-helical bundle domain of the  AAA+ domain and completely delete the peptidase domain (Fig. 1d) resulting in a loss of function of the aberrant protein.

### Clinical, electrophysiological, and neuroradiological characteristics of ALS patients with and without rare deleterious *SPG7* variants

**Fig. 2:**
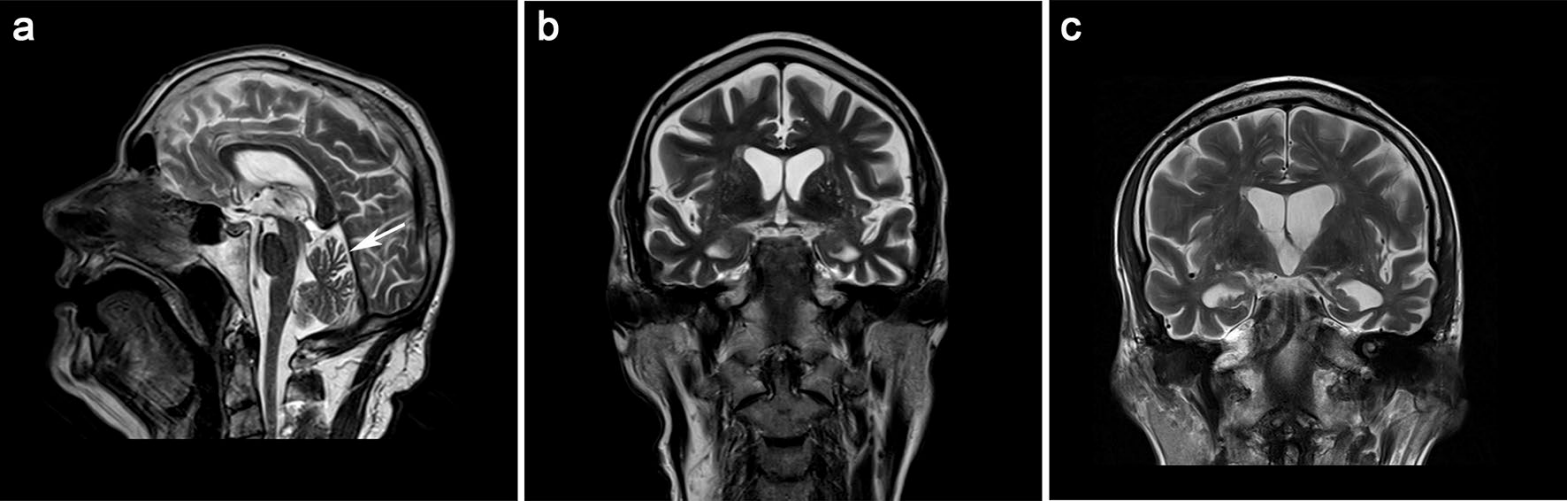
HSP- or FTD-associated patterns on brain MR images of ALS patients carrying heterozygous *SPG7* mutations. **a** Sagittal T2-weighted scan of patient VALS008 carrying the p.(R400W) variant demonstrates vermis atrophy (designated by an arrow). In addition, CC thinning was observed with a total CC area of 496 mm^2^. **b**, **c** Pronounced parietotemporal atrophy was detected in three of seven ALS patients carrying rare heterozygous *SPG7* variants, whereby coronal T2-weighted images of patient VALS008 carrying the p.(R400W) variant (**b**), and patient VALS125 carrying the p.(R486Q) variant (**c**) are shown

A summary of clinical, electrophysiological, and neuroradiological features of the ALS patients carrying rare deleterious *SPG7* variants (*n* = 9) is given in Table 2. In all *SPG7* variant carriers, acute and chronic denervation signs were detected by EMG (Table 2). NCS showed a predominantly axonal motor neuropathy in all *SPG7* variant carriers attributable to loss of motor axons in ALS, and additionally an axonal sensory neuropathy in three *SPG7* variant carriers (VALS125, MD075, MD018). The latter three and three other *SPG7* variant carriers (MD087, VALS008, VALS093) presented with pallhypaesthesia. No apparent underlying causes for the sensory impairment in these six patients such as long-term diabetes mellitus, chronic alcohol abuse, or chemotherapy were known. Brain MRI studies were available for seven patients with *SPG7* variants (Table 2). The corticospinal tract T2 hyperintensity frequently described in ALS was not detected in any of the *SPG7* variant carriers. MRI analysis showed varying degrees and patterns of brain atrophy consistent with a pattern suggestive of FTD in three cases carrying *SPG7* variants (VALS008, VALS125, VALS093) (Fig. 2). A clinical diagnosis of FTD was confirmed 1 year after ALS symptom onset for patient VALS125, who only reached 10 of 18 points in the FAB screen showing cognitive impairment in conceptualization, motor programming and executive control of action as well as inhibitory control. Patient VALS093 also displayed clinical features of FTD, i.e. verbal impairment with respect to speech and comprehension with normal memory function. No neuropsychological testing was performed in patient VALS008, who had a rapid ALSFRS-R disease progression score of 1.76. These three patients carrying *SPG7* variants also showed the most prominent CC thinning (< 500 mm^2^). The FTD-specific cognitive impairments disturbed executive function and reduced verbal fluency were identified by ECAS in the *SPG7* variant carrier MD087. Cranial MRI revealed vermis atrophy in *SPG7* variant carriers MD087 and VALS008 (Fig. 2a), and two others (TALS012-01, VALS020) showed a cerebellar atrophy pattern, clinically manifesting as intention tremor in patients VALS008 and VALS020. Cerebellar dysfunction was also clinically observed in *SPG7* variant carriers VALS125 (saccadic pursuit) with no correlating cerebellar atrophy on brain MRI at the time of investigation, TALS002-01 (ataxic gait) and MD018 (saccadic pursuit, nystagmus) with no available brain MRI. Patient VALS125, harboring the p.(R486Q) variant, and patients VALS093 and TALS002-01, both carrying the p.(A510V) variant, were diagnosed with flail arm syndrome.

**Table 2:** Clinical, electrophysiological, and neuroradiological characteristics of ALS patients carrying rare heterozygous deleterious *SPG7* variants

Patient	MD087	VALS008	TALS012-01	VALS020	VALS125	TALS002-01	MD075	VALS093	MD018
*SPG7* variant	c.1045G > A	c.1198C > T	c.1457G > A	c.1457G > A	c.1457G > A	c.1529C > T	c.1529C > T	c.1529C > T	c.1552 + 1G > T
Sex	F	M	F	M	M	M	M	M	M
Diagnosis	sALS-FL	sALS	sALS	sALS	sALS-FA	sALS-FA	sALS	sALS-FA	sALS-UMN
Age at onset, years	55	73	48	64	70	38	71	66	52
Site of onset	Spinal (LL)	Spinal (LL)	Spinal (UL)	Spinal (LL)	Spinal (UL)	Spinal (UL)	Bulbar	Spinal (UL)	Spinal (LL)
Disease duration, years^a^	3.17	1.33	7.00	5.08	2.58	9.42	1.17	3.33	11.25
Last ALSFRS-R	32	17	2	29	32	25	39	23	20
Mean ALSFRS-R-PR	0.43	1.76	0.38	0.61	0.85	0.38	0.90	0.54	0.33
Bulbar involvement	–	+	+	+	+	+	+	–	+
UL-spasticity	–	–	–	–	–	–	–	–	+
UL-DTR	↑	↑	↑	↑	↓	↑	↑	↓	↑
UL-atrophy/fasciculation	–/–	+/+	+/+	+/+	+/+	+/+	+/+	+/+	+/+
UL-weakness	+	+	+	+	+	+	+	+	+
LL-spasticity	–	–	–	–	–	–	–	–	+
LL-DTR	↑	↑	↑	↑	↑	↑	↑	↓	↑
Babinski (right/left)	–/–	–/–	–/–	–/–	–/–	+/+	–/–	+/−	+/+
LL-atrophy/fasciculation	+/−	+/+	–/+	+/+	–/+	–/ +	+/+	+/+	+/+
LL-weakness	+	+	+	+	+	+	–	+	+
Respiratory insufficiency	–	NIV	NIV	IV	–	IV	–	NIV	NIV
Pallhypaesthesia	+	+	–	–	+	–	+	+	+
Cerebellar dysfunction	–	IT	–	IT	SP	AG	–	–	SP/N
Bladder dysfunction	–	–	–	–	–	–	–	–	+
Cognitive impairment	+	n.a.	n.a.	n.a.	+	n.a.	n.a.	+	n.a.
Environmental factors	n.a.	n.a.	Smoking	n.a.	TBI	Smoking	Smoking	n.a.	Smoking
CK (U/L)	164	98	267 (↑)	398 (↑)	197 (↑)	n.a.	100	119	279 (↑)
NCS	AMN	AMN	AMN	AMN	ASMN	AMN	ASMN	AMN	ASMN
EMG	ACD	ACD	ACD	ACD	ACD	ACD	ACD	ACD	ACD
Brain MRI	+	+	+	+	+	–	+	+	–
TCC (mm^2^)	570 (↓)	496 (↓)	n.a.	542 (↓)	482 (↓)	n.a.	621	448 (↓)	n.a.
TA	–	+	–	–	+	n.a.	–	+	n.a.
Structural cerebellar abnormalities	VA	VA	CA	CA	-	n.a.	–	–	n.a.
CST	–	–	–	–	–	n.a.	–	–	n.a.

Clinical features are given that were diagnosed until the last follow-up visit

*+* present, *–* absent, *↑* increased, *↓* decreased, *ACD* acute and chronic denervation, *AG* ataxic gait, *ALSFRS-R* amyotrophic lateral sclerosis functional rating scale–revised (range 0–48 points), *ALSFRS-R-PR* ALSFRS-R-progression rate (low numbers indicate slow progression), *AMN* axonal motor neuropathy, *ASMN* axonal sensorimotor neuropathy, *CA* cerebellar atrophy, *CK* creatine kinase (increased level > 171U/L), *CST* corticospinal tract T2 hyperintensity, *DTR* deep tendon reflex, *EMG* electromyography, *F* female, *FA* flail arm, *FL* flail leg, *IT* intention tremor, *IV* invasive ventilation, *LL* lower limb, *M* male, *MRI* magnetic resonance imaging, *N* nystagmus, *n.a.* not available, *NCS* nerve conduction studies, *NIV* non-invasive ventilation, *sALS* sporadic amyotrophic lateral sclerosis, *SP* saccadic pursuit, *TA* temporal atrophy, *TBI* traumatic brain injury, *TCC* total corpus callosum area: decrease determined by comparison to anatomical references (normal range 580–1040 mm^2^) [20], *UL* upper limb, *UMN* upper motor neuron, *VA* vermis atrophy

^a^Until last follow-up (VALS020, TALS002-01, MD018) or death (MD087, VALS008, TALS012-01, VALS125, MD075, VALS093)

When comparing the characteristics in ALS patients from our cohort with (*n* = 9) and without (*n* = 205) rare deleterious *SPG7* variants (Supplementary Table 3), clinical cerebellar dysfunction was more frequent in *SGP7* variant carriers (5/9: 56%) compared to non-*SPG7* variant carriers (16/205: 8%; *P* < 0.001, two-sided Fisher’s exact test). Furthermore, *SPG7* variant carriers were overrepresented in two ALS subgroups, i.e. flail arm and flail leg syndrome, whereby flail arm syndrome was significantly more frequent in *SPG7* variant carriers (3/9: 33%) versus non-*SPG7* variant carriers (17/205: 8%; *P* = 0.04, two-sided Fisher’s exact test). However, no significant difference was detected when comparing *SPG7* variant carriers with clinical or radiological signs of FTD (4/9: 44%) and non-*SPG7* variant carriers exhibiting cognitive impairment consistent with FTD as assessed by ECAS (9/30: 30%; *P* = 0.337, two-sided Fisher’s exact test). Mean disease duration in *SPG7* variant carriers was longer, i.e. 4.9 years, than in non-carriers, i.e. 3.7 years. However, the difference in disease duration (comparing 9 *SPG7* variant carriers versus 205 non-carriers; *P* = 0.20, *T* test) and in survival (comparing 6 *SPG7* variant carriers versus 88 non-carriers; P = 0.749, log-rank test) did not reach statistical significance (Supplementary Table 3).

## Discussion

In this study, we provide evidence that rare deleterious variants in the *SPG7* gene encoding paraplegin contribute to the pathogenesis of ALS. We applied WES to a pilot cohort of 23 European ALS patients, and recurrently detected rare heterozygous *SPG7* variants previously reported in HSP or ALS. Next, by targeted sequencing in an ALS validation cohort (*n* = 191), seven further patients were identified to carry rare heterozygous *SPG7* variants known to be associated with HSP or ALS. In total, we identified heterozygous deleterious *SPG7* variants in 9 of 214 (4.2%) European ALS patients and report on the predicted consequences of these variants on the protein level as well as on the clinical, electrophysiological, and neuroradiological characteristics of the variant carriers.

Mitochondrial dysfunction and protein aggregation contribute to ALS pathogenesis [1]. As paraplegin is an AAA+ protease implicated in the degradation of misfolded proteins in mitochondria [14], a link between paraplegin and ALS is plausible making *SPG7* an attractive genetic risk factor candidate for ALS. In addition, paraplegin has structural homologies to valosin containing protein (VCP), a protein with two AAA+ domains encoded by the *VCP* gene that can cause fALS when heterozygously mutated [21]. A heterozygous *VCP* mutation was also reported in a family with complex HSP, giving evidence of overlapping molecular mechanisms in different MNDs [4]. Similarly, mutations in *SPG7* already known to cause a wide disease spectrum including autosomal recessively inherited spastic paraplegia type 7 [22], adult-onset ataxia [23], sporadic adult-onset UMN syndrome [24], chronic progressive external ophthalmoplegia [25], and autosomal dominantly inherited optic neuropathy [19], may also be linked to ALS. In addition to our data, supporting evidence for an association of *SPG7* mutations with ALS comes from a panel-based sequencing study reporting rare heterozygous deleterious variants in *SPG7* in 4 out of 80 (5%) German ALS patients [26].

**Fig. 3:**
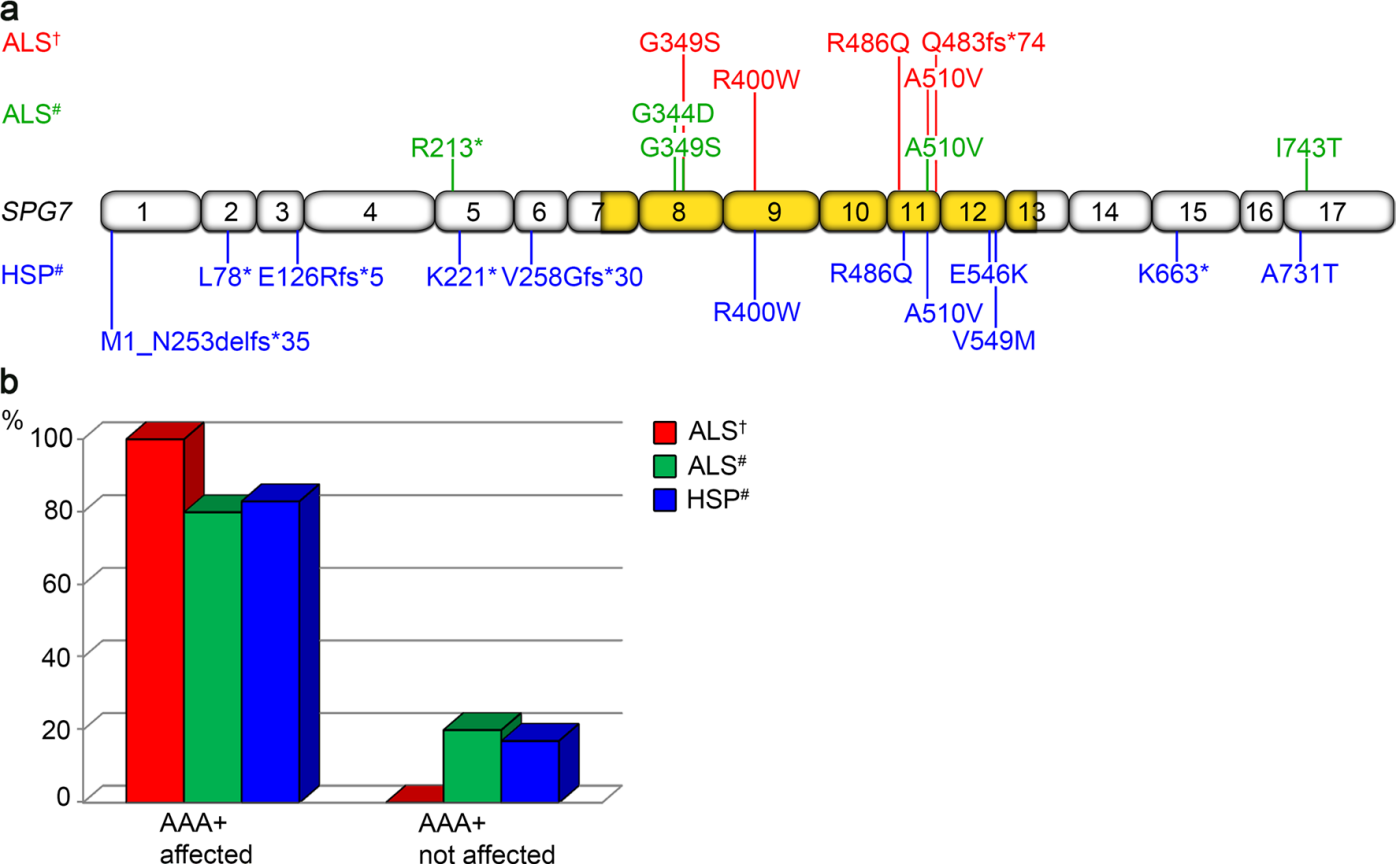
Overview of rare heterozygous deleterious *SPG7* variants reported in ALS or HSP. **a** Summary of rare [MAF < 1% in Europeans (non-Finnish) according to the ExAC database] *SPG7* variants predicted to be deleterious by at least one of two prediction tools, i.e. SIFT or PolyPhen-2, identified in ALS and HSP cases in a heterozygous state in our study and previously. The schematic illustration depicts all 17 exons of the *SPG7* gene, whereby the genomic region encoding the AAA+ domain is highlighted in gold. Variants identified in ALS patients from our cohort are given in red. Variants previously described in ALS patients [26, 27, 38] are shown in green, and variants previously reported in HSP patients [12, 19, 39, 40] are indicated in blue. Protein sequence variants are given according to the Human Genome Variation Society recommendation v15.11. The exon structure of the *SPG7* gene (NM_003119.2) was based on Alamut Visual Version 2.8. **b** The majority of rare heterozygous deleterious *SPG7* variants in ALS and HSP patients affect the  AAA+ domain that couples ATP hydrolysis to protein remodeling, i.e. 5/5 (100%) identified in ALS patients of our study, 4/5 (80%) previously described in ALS patients [26, 27, 38], and 10/12 (83%) previously detected in HSP cases [12, 19, 39, 40]. †—variants identified in our study. #—variants previously described. *AAA+ domain* ATPases associated with diverse cellular activities domain, *ALS* amyotrophic lateral sclerosis, *HSP* hereditary spastic paraplegia

All *SPG7* variants detected here in a heterozygous state in ALS patients had already been reported in ALS or HSP arguing in favor of their deleteriousness with respect to both MNDs. The pathogenicity of *SPG7* variants p.(G349S) and p.(A510V) described in ALS patients here and previously [26, 27] was further confirmed by yeast complementation assay, showing a diminished proteolytic activity of a protein complex composed of paraplegin harboring these amino acid changes and a proteolytically inactive binding partner, AFG3L2 [28]. Two other *SPG7* variants detected here in ALS patients, the p.(R400W) variant that is absent from the ExAC database (60,706 individuals) and the rare p.(R486Q) variant, were previously described as heterozygous mutations in HSP [12, 19]. The heterozygous splice site variant c.1552 + 1G > T found in an ALS patient here had been reported in an HSP patient in the homozygous state [18], and led to an abnormally spliced mRNA in both cases. Furthermore, all *SPG7* variants identified in the present study affect the highly conserved  AAA+ domain of paraplegin (Fig. 3) that couples ATP hydrolysis to protein remodeling [14]. Functional consequences can be assumed for all detected *SPG7* variants because they are predicted to destabilize the α-helical bundle, affect nucleotide binding or oligomerization, or completely lack the peptidase domain. The AAA+ domain was also affected by most rare heterozygous deleterious *SPG7* variants previously reported in both ALS and HSP (Fig. 3).

Brain MRI was reassessed in seven of nine ALS patients found to carry heterozygous *SPG7* variants here, because subtle white matter alterations in the frontal CC had previously been reported in heterozygous carriers of the *SPG7* splice site variant c.1552 + 1G > T via diffusion tensor imaging (DTI) [18]. In three of seven ALS patients, we observed CC thinning and cerebral atrophy consistent with a cortical atrophy pattern suggestive of FTD. In line with our findings, CC and cortical thinning have been reported in HSP as well as in ALS using MRI volumetry [17, 29]. Two of our patients with cerebral and CC atrophy underwent cognitive testing and showed signs of cognitive impairment suggesting a potential role of rare heterozygous *SPG7* variants in the ALS-FTD disease spectrum. Similarly, cognitive impairment in *SPG7*-associated HSP has been demonstrated in a family with three HSP patients harboring the same homozygous *SPG7* variant and in another case with HSP type 7 [30, 31]. The third patient showing CC and cerebral volumetric reduction here additionally exhibited vermis atrophy. Vermis atrophy was also diagnosed in patient MD087 who had age- and education-adjusted ALS-specific abnormalities in ECAS. Cerebellar atrophy including vermis atrophy has been reported in *SPG7*-associated HSP [19, 29], but not as a common ALS-associated feature suggesting that this alteration may be associated with ALS patients carrying rare heterozygous *SPG7* variants, although not fully penetrant. Recently, T2 hyperintensity of the dentate nucleus on brain MRI was reported in ataxia and HSP patients with biallelic *SPG7* mutations [32]. This anomaly, however, was not detected here.

Clinically and consistently with neuroradiological findings, ALS patients with rare *SPG7* variants significantly more frequently showed cerebellar dysfunction such as ataxic gait, intention tremor, and saccadic pursuit compared to ALS patients without rare *SPG7* variants (56% versus 8%). In line with our findings in ALS patients, ataxia was observed in 57% of HSP patients carrying mutations in the *SPG7* gene [30], giving further evidence for cerebellar involvement as an *SPG7* mutation-associated phenotypic feature. Pallhypaesthesia diagnosed here in six of nine ALS patients harboring rare *SPG7* variants represents another overlap with non-motor neuron clinical features characteristic of pure HSP. In addition, an overrepresentation of *SPG7* variant carriers in two rare ALS subgroups, i.e. flail arm (*P* < 0.05) and flail leg syndrome, was observed by comparison of ALS patients from our cohort with and without rare *SPG7* variants. The flail arm and flail leg phenotypes are characterized by symmetry of features and LMN predominance. Patients with flail arm and flail leg syndromes were shown to have significantly longer survival than typical ALS cases [7]. In line with these findings, there was a slight trend toward longer disease duration in *SPG7* versus non-*SPG7* variant carriers (Supplementary Table 3), suggesting that ALS patients with rare *SPG7* variants may have a somewhat better prognosis than non-carriers.

On the other hand, it is noteworthy that HSP patients mostly harboring biallelic *SPG7* variants show a better prognosis than individuals diagnosed with ALS, although the ALS patients described here carry uniallelic *SPG7* mutations only. Potential explanations for this discrepancy include an oligogenic etiology in ALS [33], suggesting that our patients may harbor additional ALS-related variants, and a dominant negative effect proposed by several studies describing heterozygous *SPG7* mutations in individuals diagnosed with HSP [12, 19]. In cells with heterozygous *SPG7* mutations, the presence of mutant paraplegin may preclude the formation of a functional hexameric ring complex [14], despite the availability of wild-type paraplegin. Furthermore, environmental risk factors, such as smoking, vigorous physical activity or trauma, have been discussed as potential disease-causing mechanisms in ALS [34]. In this context, it is noteworthy that four of nine ALS patients carrying deleterious *SPG7* variants were long-term smokers and another patient had a traumatic brain injury in the past. Previous in vitro and in vivo studies have demonstrated that mutations in paraplegin cause increased sensitivity to oxidative and environmental stress [35, 36], suggesting that the interplay of deleterious *SPG7* variants with environmental factors may be of importance in *SPG7*-associated ALS pathogenesis.

In summary, heterozygous *SPG7* mutations affecting the AAA+ domain of the encoded protein paraplegin were detected in 4.2% of European ALS patients showing partial phenotypic overlap with *SPG7*-associated HSP and with FTD, and were overrepresented in ALS patients with cerebellar dysfunction and flail arm or flail leg syndrome.

## Electronic supplementary material

Below is the link to the electronic supplementary material.

Supplementary file1 (PDF 2786 kb)
