# Supplementary material for: SPG7 mutations in amyotrophic lateral sclerosis: a genetic link to hereditary spastic paraplegia
Source: J Neurol. 2020 May 23;267(9):2732–43. doi: 10.1007/s00415-020-09861-w (PMC7419373; doi:10.1007/s00415-020-09861-w)
Supplement: Supplementary file 1 — Supplementary file1 (PDF 2786 kb) [file 415_2020_9861_MOESM1_ESM.pdf]

Supplementary material

SPG7 mutations in amyotrophic lateral sclerosis: a genetic link to hereditary spastic paraplegia

Alma Osmanovic[1,2], Maylin Widjaja[1,2], Alisa Förster[1], Julia Weder[3], Mike P. Wattjes[4], Inken Lange[2], Anastasia Sarikidi[2], Bernd Auber[1], Peter Raab[4], Anne Christians[1], Matthias Preller[3], Susanne Petri[2], Ruthild G. Weber[1]

[1]Department of Human Genetics, Hannover Medical School, Hannover, Germany

[2]Department of Neurology, Hannover Medical School, Hannover, Germany

[3]Institute for Biophysical Chemistry, Hannover Medical School, Hannover, Germany

[4]Department of Diagnostic and Interventional Neuroradiology, Hannover Medical School, Hannover, Germany

Correspondence to Dr. Ruthild G. Weber, Hannover Medical School, Department of Human Genetics, Carl-Neuberg-Straße 1, 30625 Hannover, Germany, Phone: +49 511 532 7751, Fax: +49 511 532 18520, Email: weber.ruthild@mh-hannover.de

Alma Osmanovic and Maylin Widjaja contributed equally as first authors to this work.

Susanne Petri and Ruthild G. Weber contributed equally as senior authors to this work.

Supplementary Table 1 Analysis of whole-exome sequencing germline data from 23 ALS patients using a candidate gene-based strategy to identify ALS-associated genes

**Filtering steps**	**No. of variants**	**No. of genes**
Total variants in exomes from whole blood of 23 ALS patients	109,668	18,820
Variants with the following quality scores: coverage ≥20, call quality ≥50, and allele fraction ≥40% were retained	90,719	18,175
Population filtering: rare or novel variants (<1% according to the “ExAC Browser”) were retained	15,210	8,874
Variants classified as “pathogenic” or “likely pathogenic” according to “ACMG guidelines” were retained	87	80
Variants within ALS-associated genes according to “ALSoD” (n=126) were retained	3	2 (*SPG7*, *LIPC*)

ACMG guidelines, interpretation of sequence variants according to the American College of Medical Genetics and Genomics (Richards et al., 2015); ALS, amyotrophic lateral sclerosis; ALSoD, ALS Online Genetics Database (http://alsod.iop.kcl.ac.uk); ExAC Browser, Exome Aggregation Consortium Browser (Beta) (http://exac.broadinstitute.org/)

Reference: Richards S, Aziz N, Bale S, Bick D, Das S, Gastier-Foster J, Grody WW, Hegde M, Lyon E, Spector E, Voelkerding K, Rehm HL, Committee ALQA (2015) Standards and guidelines for the interpretation of sequence variants: a joint consensus recommendation of the American College of Medical Genetics and Genomics and the Association for Molecular Pathology. Genet Med 17 (5):405-424. https://doi.org/10.1038/gim.2015.30

Supplementary Table 2 All variants identified in the coding and adjacent splice site (± 20 base pairs) regions of the *SPG7* gene in our cohort of 214 European ALS patients

Exon/ Intron	Variant	Chromosomal position according to GRCh37	dbSNP	MAF	Prediction	
					SIFT	PolyPhen-2
1	c.21_23dupGCT p.(L8dup)	16:89574846_ 89574848	rs781285980	-	-	-
	c.120G>A p.(G40G)	16:89574945	rs187330648	0.01476	-	-
4	c.447G>A p.(A149A)	16:89590484	rs1164011433	-	-	-
	c.618+12T>C p.(?)	16:89590667	rs3803679	0.4568	-	-
5	c.730T>G p.(S244A)	16:89592848	rs527731763	0.00004495	Tolerated	Benign
6	c.795G>A p.(L265L)	16:89595921	-	-	-	-
7	c.987+5A>G p.(?)	16:89597221	rs4785691	0.4536	-	-
8	c.1032C>T p.(G344G)	16:89598356	rs116319889	0.007308	-	-
	c.1045G>A p.(G349S)	16:89598369	rs141659620	0.001437	Deleterious	Probably damaging
9	c.1198C>T p.(R400W)	16:89598918	rs748024868	-	Deleterious	Probably damaging
10	c.1449+19G>A p.(?)	16:89611199	rs79201073	0.06351	-	-
11	c.1457G>A p.(R486Q)	16:89613073	rs111475461	0.007232	Deleterious	Benign
	c.1507A>G p.(T503A)	16:89613123	rs2292954	0.189	Tolerated	Benign
	c.1529C>T p.(A510V)	16:89613145	rs61755320	0.004004	Deleterious	Probably damaging
	c.1552+1G>T p.(Q483fs*74)	16:89613169	rs141644720	0.00006002	-	-
12	c.1663+13C>T p.(?)	16:89614534	rs80324518	0.09108	-	-
14	c.1830C>T p.(L610L)	16:89619437	rs746099594	0.00006072	-	-
	c.1936+16G>A p.(?)	16:89619559	rs748678461	0.0002725	-	-
	c.1937-16C>G p.(?)	16:89620186	rs74590011	0.002374	-	-
15	c.2063G>A p.(R688Q)	16:89620328	rs12960	0.1895	Tolerated	Possibly damaging
17	c.2280G>A p.(P760P)	16:89623393	rs11559075	0.01513	-	-
	c.2292C>T p.(I764I)	16:89623405	rs61747711	0.0451	-	-

dbSNP, Single Nucleotide Polymorphism database; MAF, minor allele frequency in European (non-Finnish) population according to Exome Aggregation Consortium Browser (Beta); SIFT according to Alamut VISUAL version 2.11.
Reference sequence used: NM_003119.2.

Supplementary Table 3 Comparison of characteristics in ALS patients with and without rare heterozygous deleterious *SPG7* variants

**Characteristics**	***SPG7* variant carriers (n=9):** number (%) or mean (range)	**Non-*SPG7* variant carriers (n=205):** number (%) or mean (range)	**P value**
**Inheritance**			
Familial	0 (0)	6 (2.9)	1
Sporadic	9 (100)	199 (97.1)	1
**Sex**			
Male	7 (77.8)	119 (58.05)	0.31
Female	2 (22.2)	86 (41.95)	0.31
**Age at onset, y**	59.7 (38-73)	59.9 (25-84)	0.94
**Disease duration+, y**	4.9 (1.2-11.3)	3.7 (0.4-18.0)	0.20
**Survival, y**	5.7 (1.2-7.0)	7.7 (0.4-17.1)	0.749
**Site of onset**			
Bulbar	1 (11.1)	49 (23.9)	0.69
Spinal	8 (88.9)	156 (76.1)	0.69
**ALS subgroup**			
Classic ALS	4 (44.4)	140 (68.3)	0.16
UMN	1 (11.1)	6 (2.9)	0.26
LMN	0 (0)	16 (7.8)	1
Flail arm	3 (33.3)	17 (8.3)	**0.04***
Flail leg	1 (11.1)	1 (0.5)	0.08
PMA	0 (0)	4 (2.0)	1
Bulbar	0 (0)	19 (9.3)	1
Respiratory	0 (0)	2 (0.9)	1
**ALSFRS-R score at first visit**	37.7 (24-45)	38.7 (6-47)#	0.60
**ALSFRS-R progression rate**	0.55 (0.13-1.25)	0.65 (0.04-3.00)#	0.61
**FTD-specific findings**	4 (44)	9 (30)†	0.337
**Cerebellar dysfunction**	5 (55.6)	16 (7.8)	**0.00055***

+Until last follow-up or death

#ALSFRS-R scores and progression rates were not available from 17 non-*SPG7* variant carriers

†Tested by ECAS in 30 non-*SPG7* variant carriers with age- and education-adjusted cut offs

*Significant difference at $p<0.05$. Comparisons between *SPG7* variant carriers and non-carriers were made using T-test for continuous variables or Fisher's exact test (two-sided) for dichotomous variables. Survival analysis was performed by log-rank test (*SPG7* variant carriers: n=6; non-*SPG7* variant carriers: n=88)

ALS, amyotrophic lateral sclerosis; ALSFRS-R, amyotrophic lateral sclerosis functional rating scale - revised; FTD, frontotemporal dementia; LMN, lower motor neuron; PMA, progressive muscular atrophy; UMN, upper motor neuron; y, years.

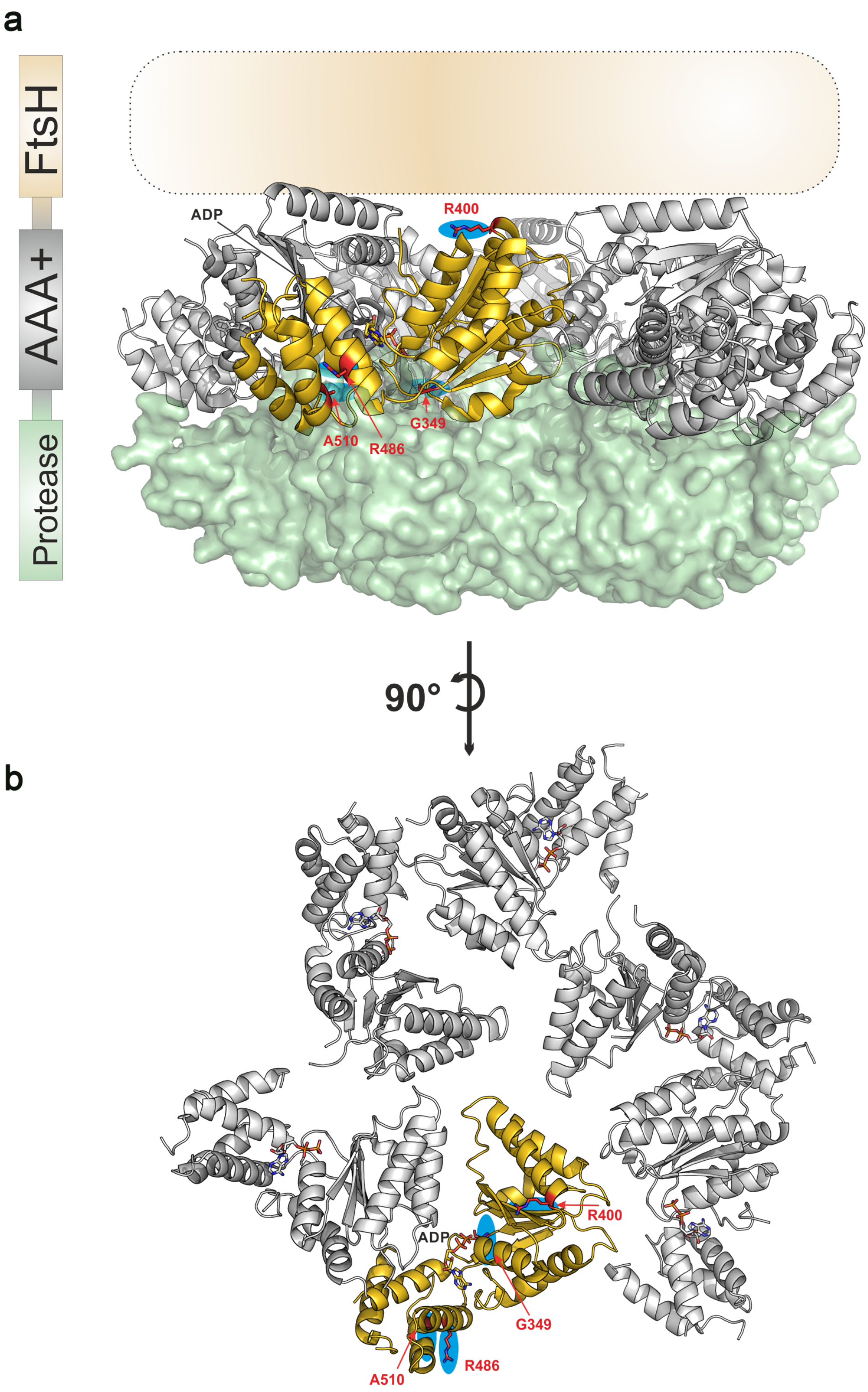

Supplementary Fig. 1 Side (**a**) and top (**b**) views of the structural model of the paraplegin complex illustrating the domain architecture. Each monomer in the hexameric complex comprises three different domains, the FtsH domain (brown), the AAA+ ATPase domain (grey), and the protease domain (green), which form overlying ring-like structures. The AAA+ domain of one monomer is coloured yellow, the positions of amino acids affected by paraplegin mutations in ALS patients are highlighted in blue, and stick representations are given. Mutations p.R400W, p.R486Q, and p.A510V are located in close vicinity to the interfaces between the AAA+ monomers or the protease domain, and may thus interfere with complex formation.
